# Alterations of amygdala volume and functional connectivity in migraine patients comorbid with and without depression

**DOI:** 10.1002/brb3.3427

**Published:** 2024-02-15

**Authors:** Xin Chen, Wei Gui, Han‐Li Li, Zi‐Ru Deng, Yu Wang

**Affiliations:** ^1^ Department of Neurology First Affiliated Hospital of Anhui Medical University Hefei China; ^2^ Department of Neurology Anhui Public Health Clinical Center Hefei China; ^3^ Department of Neurology First Affiliated Hospital of University of Science and Technology of China Hefei China; ^4^ Department of Neurology, Epilepsy and Headache Group First Affiliated Hospital of Anhui Medical University Hefei China

**Keywords:** amygdala, centromedial amygdala, depression, functional connectivity, migraine, voxel‐based morphometry

## Abstract

**Objective:**

The comorbid relationship between migraine and depression has been well recognized, but its underlying pathophysiology is unclear. Here, we aimed to explore the structural changes of the amygdala and the abnormal functional connectivity of the centromedial amygdala (CMA) in migraineurs with depression.

**Methods:**

High‐resolution T1‐weighted and functional magnetic resonance images were acquired from 22 episodic migraineurs with comorbid depression (EMwD), 21 episodic migraineurs without depression (EM), and 17 healthy controls (HC). Voxel‐based morphometry and resting‐state functional connectivity (rsFC) were applied to examine the intergroup differences in amygdala volume.

**Results:**

The bilateral amygdala volume was increased in the EMwD and EM groups compared with the HC group, but there were no differences between the EMwD and EM groups. The right CMA exhibited decreased rsFC in the left dorsolateral prefrontal cortex (DLPFC) in the EMwD group compared with the EM group, while rsFC increased between the CMA and the contralateral DLPFC in the EM group compared with the HC group. In addition, the EM group showed decreased rsFC between the left CMA and the left pallidum compared with the HC group.

**Conclusions:**

Enlarged amygdala is an imaging feature of EM and EMwD. The inconsistency of rsFC between CMA and DLPFC between migraineurs with and without depression might indicate that decreased rsFC between CMA and DLPFC is a neuropathologic marker for the comorbidity of migraine and depression. The core regions might be a potential intervention target for the treatment of EMwD in the future.

## Graphical abstract

We aimed to explore the structural change of amygdala and abnormal function in the centromedial amygdala among migraineurs with and without depression.

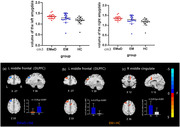

## 1 INTRODUCTION

Migraine is a recurrent headache afflicting about 10% of the population worldwide (GBD 2015 Disease & Injury Incidence & Prevalence Collaborators, 2016; Lj et al., 2007), and many migraineurs suffer from headaches and comorbidities, including depression. A 2018 review of the association between migraine and psychiatric comorbidities showed that patients with migraine are two to four times more likely to have depression than healthy people (Bergman‐Bock, 2018). Vice versa, depression facilitates the activities of migraine headaches (Schramm et al., 2021). Therefore, a deeper understanding of the neurophysiological mechanisms associated with the comorbidities of migraine and depression deserves further investigation.

Previous studies of migraines were mainly focused on the neurovascular mechanism. In recent years, the neural dysfunction model of the pain network has expanded the traditional concept of migraine (Dodick, 2018), help clarify migraine headache attacks, chronicity, and refractoriness (Maizels et al., 2012). The lifetime risk of depression in migraineurs is 80% (Leo & Singh, 2016), and such patients are more prone to anxiety and suicidal tendencies (Amoozegar, 2017). Especially in women, depression can lead to recurrent attacks of migraine and may even evolve into chronic migraine (Sair et al., 2020). A survey suggests that major depressive disorder (MDD) not only increases the frequency of attacks in migraine without aura, but also plays a variable role in different subtypes of migraine (Pisanu et al., 2020). In an animal experiment, it has been shown that neonatal maternal deprivation and chronic unpredictable stress exacerbate the development of migraines, with a greater impact observed in females (Raoof et al., 2022).

MDD is a leading global cause of disability and is accompanied by intellectual/academic impairment, alcohol/drug abuse, decreased productivity, poor appetite and nutrition disorders, impaired sleep, and general self‐destructive patterns (Knight & Baune, 2018). Migraine with MDD is a condition in which the patients will experience various extents of the symptoms of both diseases (Bergman‐Bock, 2018). A study revealed that patients with MDD have a two‐fold risk of migraine compared with non‐MDD individuals (Castelnuovo et al., 2016). The coexistence of migraine and depression constitutes the complexity of treatment, and more research on the comorbid mechanisms appears to be worth considering (Wachowska et al., 2023). There are differences in brain volume among patients with MDD and migraine (Ma et al., 2018). Migraineurs with depression might represent a distinct clinical phenotype as a brain volume study showed that migraine in combination with MDD is associated with smaller brain volume than having one or neither of these conditions (Gudmundsson et al., 2013). These changes in brain volume reflect changes in various structures that might be involved in the pathogenesis of migraine and MDD. The functions of the limbic system are involved in pain modulation, memory, motivation formation, and emotion processing (Herman et al., 2005). The amygdala, a subcortical center of the limbic system, is a key structure involved in the emotional responses to pain (Neugebauer et al., 2004; Tye et al., 2011). The amygdala plays a key role in processing negative emotions (Morris et al., 1996) and often presents structural and functional impairment in MDD (Cullen et al., 2014; Nolan et al., 2020). The amygdala can be divided into three subregions: centromedial amygdala (CMA), interconnected later basal amygdala, and superficial amygdala (Bzdok et al., 2013). The structural and functional changes in the different amygdala subregions have been observed in MDD patients (Leaver et al., 2018; Qiu et al., 2018). The CMA, known as the “nociceptive amygdala,” is the main output nucleus of the amygdala function (Neugebauer et al., 2004). The CMA has extensive connections with the forebrain cortex and brainstem related to the mediation of fear and other emotions (Han et al., 2015; Penzo et al., 2015). A resting‐state functional magnetic resonance study showed that the connectivity between the bilateral amygdala and anterior insula was increased in migraine patients (irrespective of aura) compared with healthy controls (HC) (Hadjikhani et al., 2013). One study showed increased amygdala connectivity in patients with episodic migraines without depression (EM) and was related to sleep quality (Chen et al., 2017). The resting‐state functional connectivity (rsFC) between the CMA and the dorsal raphe nucleus is decreased in patients with chronic pain and depression but not in patients without depression (Zhou et al., 2019). Life stress within the last six months was associated with smaller left amygdala volume (Sublette et al., 2016). In many animal experiments, the amygdale and the periaqueductal gray matter (GM) have also been shown involved in regulating the behavior of migraine. A study suggests an emerging role of the basolateral amygdala in regulating cognitive decline in coexisting migraine headaches in rats (Askari‐Zahabi et al., 2021). One study suggests that the ventrolateral periaqueductal GM is involved in modulating anxiety‐like behaviors and social dysfunction in migraineur rats (Pourrahimi et al., 2021). These previous studies demonstrated structural and functional alterations in migraine as well as in emotional disorders, including depression.

Because of the important role of the amygdala in migraine and depression, the motivation of our study was to investigate the structural changes and the functional connectivity (FC) of the amygdala in episodic migraine with depression (EMwD), EM without depression, and HC.

## 2 METHODS

### 2.1 Participants

The participants were recruited from the Departments of Neurology of the First Affiliated Hospital of Anhui Medical University and First Affiliated Hospital of Science and Technology University of China between March 2019 and October 2020. Migraine was diagnosed using ICHD‐3 beta (GBD 2015 Disease & Injury Incidence & Prevalence Collaborators, 2013). Depression was diagnosed by two experienced psychiatrists using the DSM‐5 criteria (Regier et al., 2013). All participants were (1) Han nationality (the main ethnicity in China), (2) right‐handed, and (3) 20−55 years old. The exclusion criteria were (1) a history of systemic diseases (e.g., secondary headache, tension headache, or chronic migraine) and severe neurological diseases (e.g., schizophrenia, mania, or bipolar disorder), (2) medication for migraine prevention in the past 3 months or overuse of painkillers, (3) intracranial lesions, or (4) contraindications to MRI. This study was approved by the Research Ethics Committee of the First Affiliated Hospital of Anhui Medical University (approval #20200470) and all participants provided written informed consent before any study procedure.

### 2.2 The EMwD group

Based on the ICHD‐3 beta and DSM‐5 criteria, 22 patients were diagnosed with EM (without aura) and MDD. They were in the stage of depressive episodes. They did not use any antidepressants or drugs to prevent migraines in the past 3 months. All patients were headache‐free for at least 3 days before and after the examination.

### 2.3 The EM group

Twenty‐one patients were diagnosed with EM (without aura) and without any history of mental illness. They had no history of medication overuse and had no prophylactic or chronic medication for at least 3 months. This group of patients had no headache for at least 3 days before and after the examination.

### 2.4 The HC group

Seventeen healthy right‐handed participants were involved. They had no history of headaches and mental illness. They had no migraine‐like headaches and no family history of headaches. A history of a chronic disease led to exclusion.

All participants completed a standardized questionnaire to determine demographic characteristics, including age, sex, and education years. In the patient groups, the Visual Analogue Scale (VAS), the Migraine Disability Assessment questionnaire (MIDAS), and the Headache Impact Test‐6 (HIT‐6) were used to assess migraine severity. The Hamilton Anxiety Scale (HAMA) and Hamilton Depression Scale (HAMD), and Mini‐mental State Examination (MMSE) were obtained from all participants.

### 2.5 MRI scanning

Images were acquired using a 3.0‐T MRI system (Discovery GE750w; GE Healthcare) with an 8‐channel head coil at the University of Science and Technology of China, Hefei, Anhui Province. During scanning, all participants closed their eyes and stayed awake. First, high‐resolution 3D structural images were acquired (repetition time [TR] = 8.16 ms; echo time [TE] = 3.18 ms; flip angle [FA] = 12°; field of view [FOV] = 2.56 × 2.56 cm; slice thickness = 1 mm, no gap; 188 sagittal slices per participant; matrix = 256×256; acquisition time = 296 s). Then, resting‐state blood‐oxygen‐level‐dependent data were obtained by using a gradient echo planar imaging sequence (TR = 2.4 s; TE = 30 ms; FA = 90°; FOV = 1.92 × 1.92 cm; matrix = 64 × 64; slice thickness = 3 mm, slice gap = 1 mm; 46 interleaved axial slices; voxel size = 3×3×3 mm; acquisition time = 370 s).

### 2.6 Amygdala volume quantification

**FIGURE 1:**
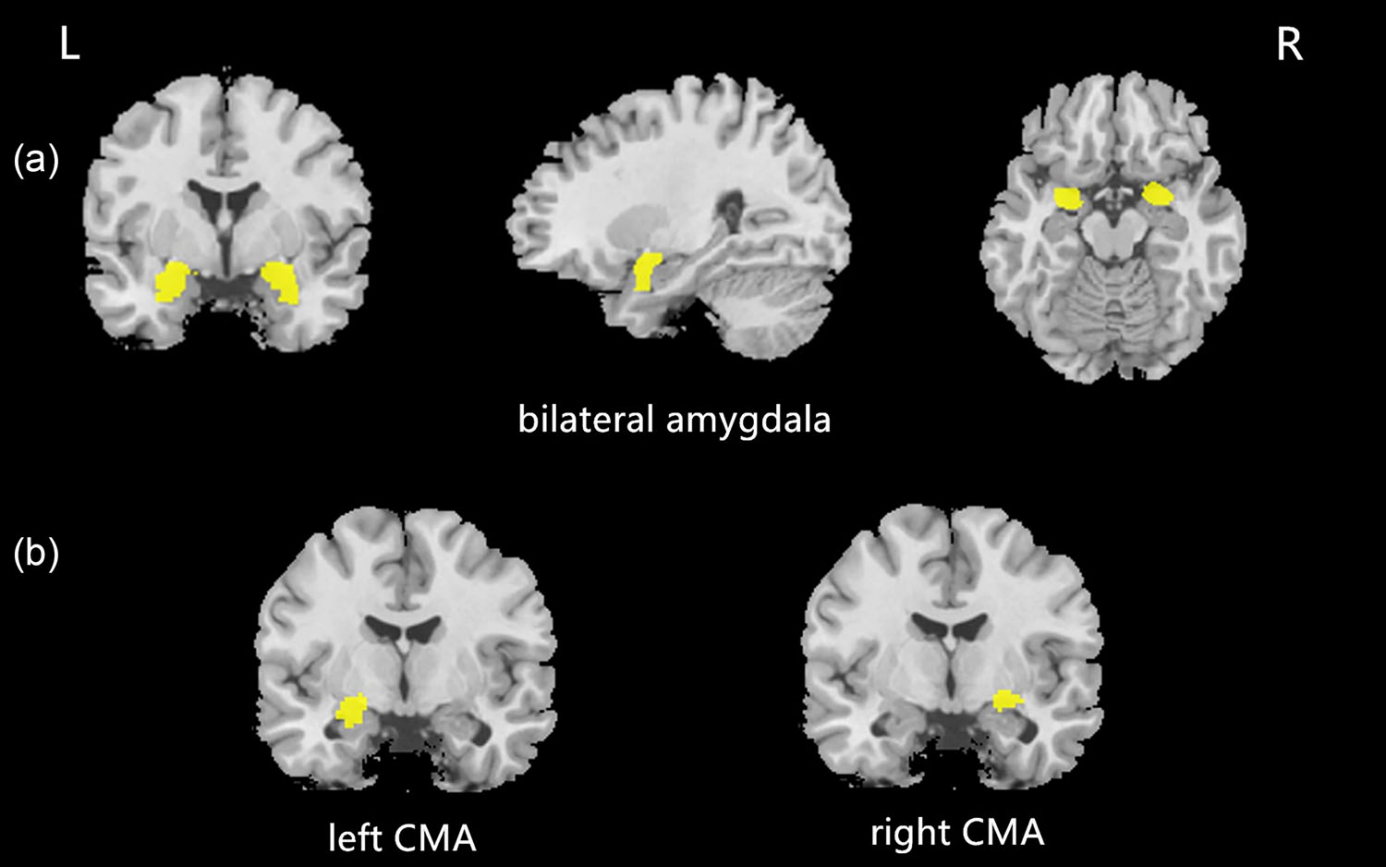
(a) The mask of the bilateral AMY; (b) the mask of the left and right CMA. AMY, amygdala; CMA, centromedial amygdala.

We used a seed‐based method to compare the amygdala volume and intrinsic FC among the EMwD, EM, and HC groups. The 3D T1‐weighted structural images were processed using SPM8 (http://www.fil.ion.ucl.ac.uk/spm) and VBM8 (http://dbm.neuro.uni‐jena.de/vbm) under MATLAB R2013b (The Mathworks), involving spatial normalization, segmentation, modulation, and smoothing. The images were segmented into GM, white matter (WM), and cerebrospinal fluid (CSF). Total intracranial volume (TIV) was the total of GM, WM, and CSF volumes. The volume of the amygdala was calculated by extracting mean voxel values in a region of interest (ROI), as defined by the AAL brain atlas, using WFU PickAtlas software as described in previous studies (Foell et al., 2019; Wei et al., 2020) (http://www.ansir.wfubmc.edu) (Figure 1a).

### 2.7 fMRI data preprocessing and FC analysis

The functional images were preprocessed using the Data Processing and Analysis for Brain Imaging software (DPARSFA, http://rfmri.org/DPARSF) in MATLAB. The first 10 volumes for each subject were discarded to avoid scanner instability. Then, the following steps were carried out: slicing time, realignment for head motion correction (all head movements exceeding 2.0 mm were excluded), spatial normalization to the Montreal Neurological Institute coordinate space (with 3×3×3 mm (Bergman‐Bock, 2018)), and smoothing with a Gaussian kernel of 6‐mm full width half maximum (FWHM). Linear trend removal and temporal band‐pass filtering (0.01−0.08 Hz) were performed.

A seed‐based analysis with the CMA was performed to explore the FC alterations. The FC analysis was performed as follows: (1) The CMA ROI mask was created from a data‐driven characterization using coactivation‐based parcellation (Bzdok et al., 2013) (Figure 1b). (2) FC of the left and right CMA was analyzed using DPARSFA. The averaged time‐courses of the ROIs were extracted. We calculated Pearson's correlations of the mean time‐courses of ROI with each voxel of the whole brain. Each voxel was 1×1×1 mm. Then, Fisher's r‐to‐z transform was conducted.

### 2.8 Statistical analysis

Continuous variables were tested for normal distribution using the Shapiro−Wilk test. Demographic variables (age, sex, education years) and clinical features (HAMA, HAMD) were analyzed using a one‐way analysis of covariance (ANCOVA) among the three groups. Then, LSD was performed to identify differences between every two groups. VAS, MIDAS, and HIT‐6 were analyzed using a two‐sample *t*‐test. The differences in amygdala volumes were compared using ANOVA using the age, sex, education years, and TIV as covariates. Statistical analyses were performed using IBM SPSS 24.0 (IBM). *p* < .05 was considered statistically significant. RsFC among the three groups was performed by ANOVA tests, while the Bonferroni method was used for pairwise comparison using the dpabi software (Yan et al., 2016). We put age, sex, education years, and the bilateral AMY volumes as the covariates. All statistical maps were corrected with a Gaussian Random Field (GRF) method. The threshold was set at *p* < .001 (voxel‐level) or *p* < .05 (cluster‐level), two‐tailed.

## 3 RESULTS

### 3.1 Demographic and clinical data

Subject demographics and neurophysiological characteristics are shown in Table 1. There were no statistically significant differences among the three groups regarding age, sex, and education years.

**TABLE 1:** The demographic and clinical characteristics of participants.

	EMwD	EM	HC	*p*‐value
Demographic and clinical data				
Sex (female/male)	22 (18/4)	21 (17/4)	17(14/3)	.994^a^
Age (years)	30.8 ± 6.8	32.2 ± 7.8	31.6 ± 9.2	.840^b^
Education years	12.4 ± 4.6	14.3 ± 3.5	14.2 ± 4.3	.254^b^
Duration of illness (years)	8.2 ± 5.5	9.1 ± 5.1	/	.584^b^
Frequency (attack‐days per month)	2.4 ± 1.7	2.2 ± 1.4	/	.732^c^
Attack duration (h)	15.4 ± 6.7	15.7 ± 7.3	/	.836^c^
VAS	6.2 ± 1.2	6.3 ± 1.5	/	.801^c^
MIDAS	23.5 ± 20.1	16.9 ± 11.6	/	.198^c^
HIT‐6	60.7 ± 4.8	59.5 ± 5.6	/	.433^c^
17‐HAMD	17.3 ± 3.9^a&^	4.0 ± 1.9^$^	1.7 ± 2.1	<.001^b^
14‐HAMA	9.99 ± 5^a&^	4.0 ± 2.7	2.4 ± 2.6	<.001^b^

*Note*: The data are shown as mean ± SD.

Abbreviations: EM, episodic migraine; EMwD, episodic migraine with depression; HAMA, Hamilton Rating Scale for Anxiety; HAMD, Hamilton Rating Scale for Depression; HC, healthy controls; HIT‐6, Headache Impact Test; MIDAS, Migraine Disability Assessment questionnaire; VAS, visual analog scale.

^a^ Pearson Chi‐square test.

^b^ One‐way ANCOVA tests.

^c^ Two‐sample *t*‐tests.

^a^ *p* < .001 versus EM; ^&^ *p* < .001 versus HC; ^$^ *p* < .05 versus HC.

### 3.2 Intergroup differences in amygdala volume

**FIGURE 2:**
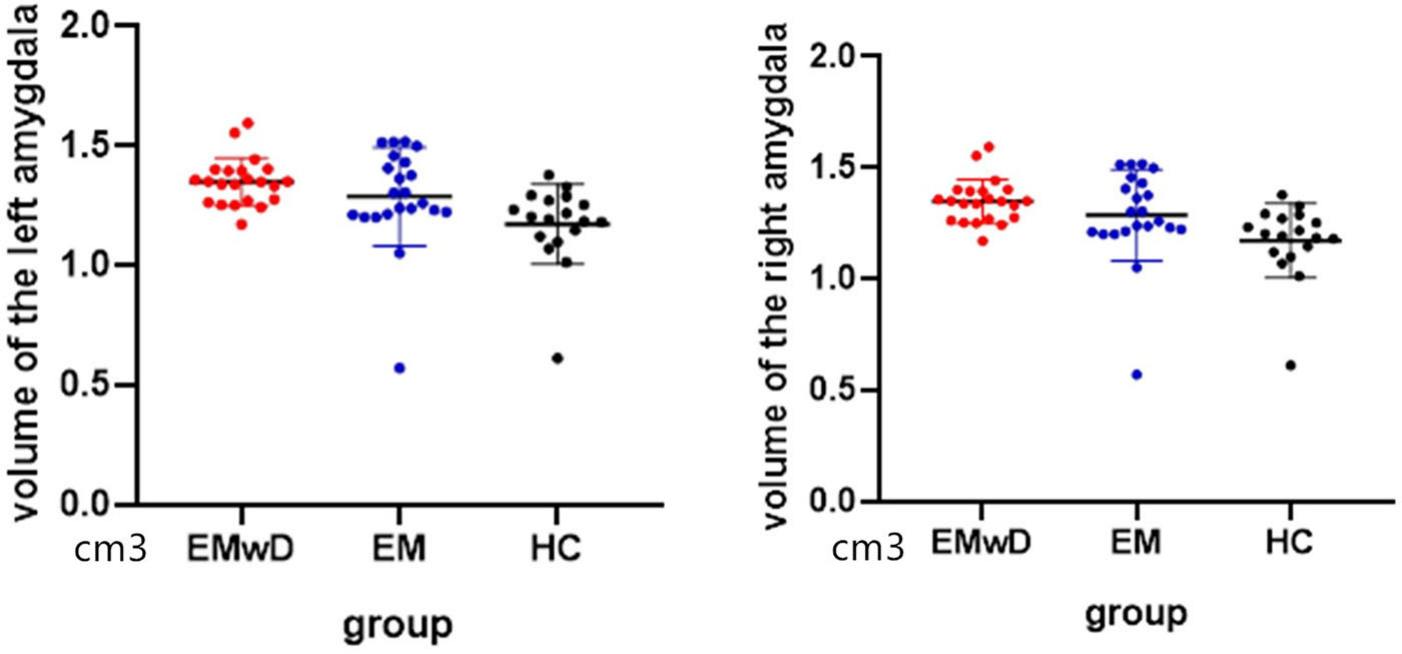
AMY volume of the three groups. The Y‐axis indicates the volume of amygdala. AMY, amygdala; EM, episodic migraine; EMwD, migraine comorbid with depression; HC, healthy controls.

The volume of the amygdala, GM, WM, and total intracranial are shown in Table 2. Figure 2 shows the left and right amygdala volumes of the three groups. The comparisons of the bilateral amygdala volumes among the three groups are presented in Table 3. The results revealed significant differences in the volume between EMwD and HC and between EM and HC (all *p* < .01). Compared with the HC group, the amygdala volume was increased in the EMwD and EM groups. Our study did not reveal any significant difference in amygdala volume between the EMwD and EM groups.

**TABLE 2:** Volumes of patients and healthy controls.

Volumes (cm^3^)	EMwD (*n* = 22)	EM (*n* = 21)	HC (*n* = 17)	*p*‐value
L‐amygdala	1.28 ± 0.1^&^	1.25 ± 0.13^$^	1.14 ± 0.09	
R‐amygdala	1.34 ± 0.1^&^	1.32 ± 0.13^$^	1.20 ± 0.1	
Gray matter	634.05 ± 53.33	657.57 ± 53.74	663.44 ± 58.35	.205
White matter	527.08 ± 58.55	545.16 ± 52.48	539.64 ± 52.06	.544
TIV	1369.09 ± 127.29	1421.59 ± 116.87	1422.24 ± 122.17	.281

*Note*: Data are presented as mean ± SD. The *p*‐value was separate one‐way ANCOVA tests. Post‐hoc analyses were conducted using the LSD method.

Abbreviations: EM, episodic migraine; EMwD, episodic migraine with depression; HC, healthy controls; L, left; R, right; TIV, total intracranial volume.

& *p* < .001 versus HC; $ *p* < .05 versus HC.

**TABLE 3:** The comparison of amygdala volume among groups using ANCOVA.

	Mean difference (95% CI)^*^	Std. error.	Sig.^a^
EMwD versus EM (left/right)	0.0279 (−0.0381 to 0.0914)/ 0.0277 (−0.0399 to 0.0953)	0.0326/0.0338	0.413/0.416
EMwD versus HC (left/right)	0.1411 (0.0726−0.2097)/	0.0342/0.0357	<0.001/ < 0.001
	0.1452 (0.0737−0.2168)		
EM versus HC (left/right)	0.1145 (0.0452−0.1837)/ 0.1176 (0.0453−0.1899)	0.0346/0.0361	0.002/0.002

Abbreviations: CI, confidence interval; EM, episodic migraine; EMwD, migraine comorbid with depression; HC, healthy controls.

* The mean difference is significant at the 0.05 level.

^a^ Adjustment for multiple comparisons: Least Significant Difference (equivalent to no adjustments).

### 3.3 Intergroup differences in rsFC of CMA

**FIGURE 3:**
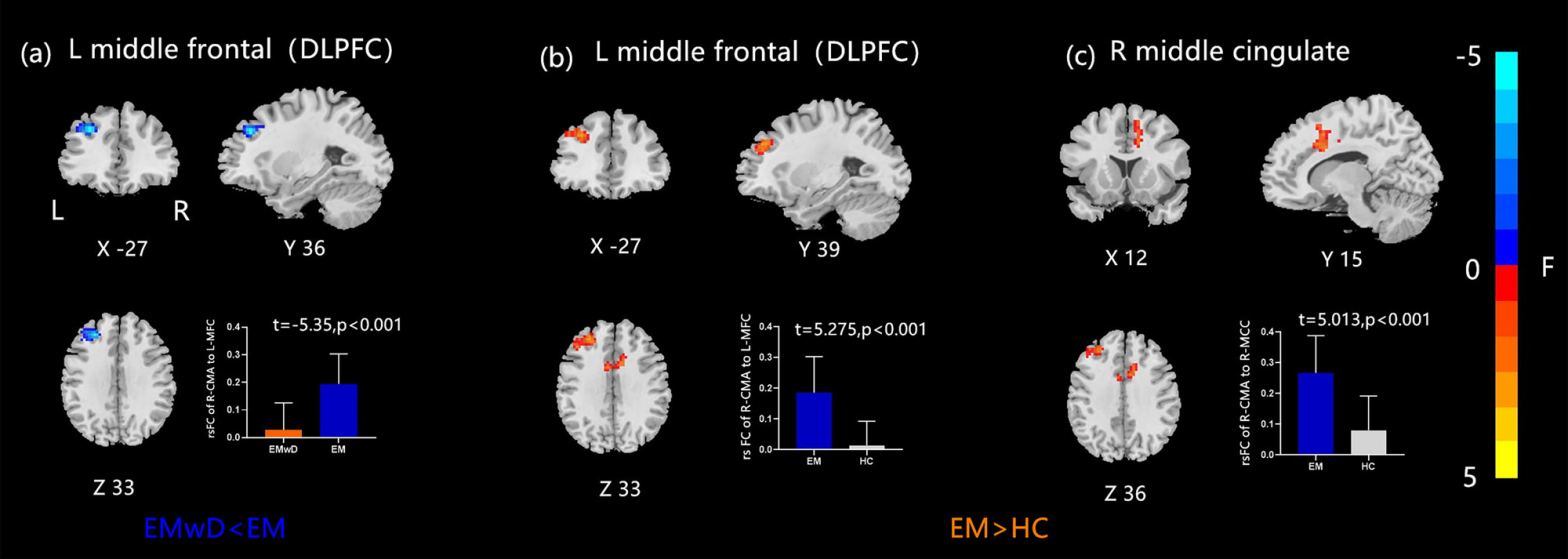
Altered effective connectivity between the right CMA and whole brain regions in three groups. (a) EMwD versus EM: decreased rsFC in the left DLPFC; (b) EM versus HC: increased rsFC in the left DLPFC and right middle cingulate. CMA, centromedial amygdala; DLPFC, dorsolateral prefrontal cortex; EM, episodic migraine; EMwD, migraine comorbid with depression; HC, healthy controls; rsFC, resting‐state functional connectivity.

**FIGURE 4:**
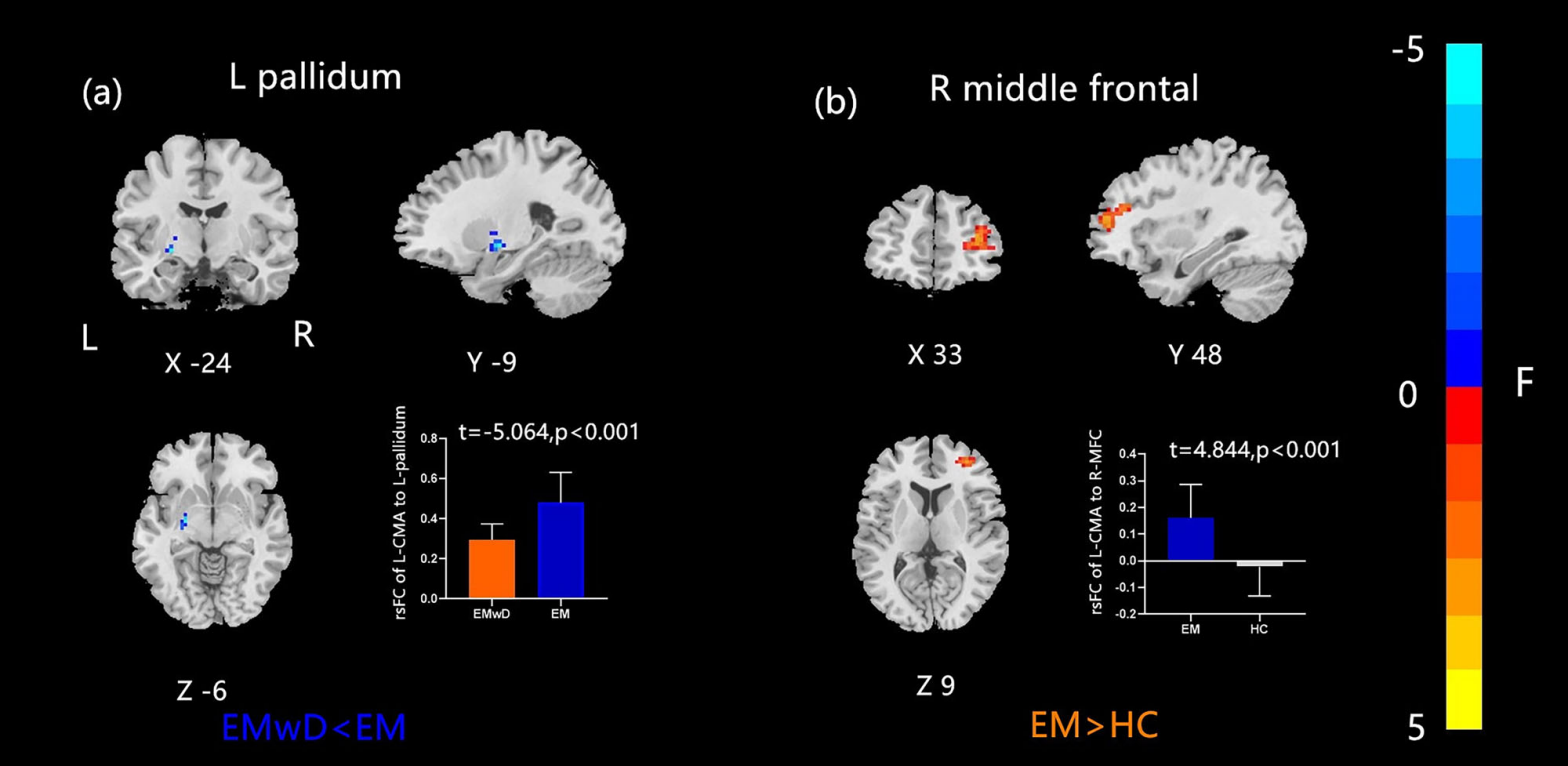
Altered effective connectivity between the left CMA and whole brain regions in three groups. (a) EMwD versus EM: decreased rsFC in the left pallidum; (b) EM versus HC: increased rsFC in the right DLPFC. CMA, centromedial amygdala; DLPFC, dorsolateral prefrontal cortex; EM, episodic migraine; EMwD, migraine comorbid with depression; HC, healthy controls; rsFC, resting‐state functional connectivity.

Compared with the EM group, the EMwD group showed a decreased rsFC between the right CMA and left dorsolateral prefrontal cortex (DLPFC). Compared with the HC group, the EM group showed a significantly increased rsFC of the right CMA with the left DLPFC, as well as with the right middle cingulum (Figure 3 and Table 4). For the left CMA, the EMwD group showed a decreased rsFC with the left pallidum compared with the EM group, and the EM group presented an increased rsFC with the right middle frontal cortex compared with the HC group (Figure 4 and Table 4).

**TABLE 4:** Abnormal functional connectivity of the bilateral centromedial amygdala among the three groups.

Seed	Group	Region (AAL)	Region (BA)	MNI			Number of voxels	Peak *t*
				x	y	z		
L‐CMA	EMwD versus EM	Pallidum_L		−24	−9	−6	22	−3.9901
	EM versus HC	Frontal_Mid_R	45,46	33	48	9	83	4.1979
R‐CMA	EMwD versus EM	Frontal_Mid_L	9,46	−27	36	33	76	−4.6819
	EM versus HC	Frontal_Mid_L	9,46	−27	39	33	85	4.84
		Cingulum_Mid_R	32	12	15	36	66	4.1037

*Note*: Voxel level set at *p* < .001. Cluster level set at *p* < .05 (GRF correction).

Abbreviations: BA, Brodmann area; CMA, centromedial amygdala; L, left; MNI, Montreal Neurological Institute; R, right.

## 4 DISCUSSION

This study aimed to explore the structural changes of the amygdala and the abnormal FC of the CMA in migraineurs with depression. The results suggest that an enlarged amygdala is an imaging feature of EM and EMwD. The inconsistency of rsFC between CMA and DLPFC between migraineurs with and without depression might indicate that decreased rsFC between CMA and DLPFC is a neuropathologic marker for the comorbidity of migraine and depression.

In this study, we computed the amygdala volume by extracting the mean voxel values in the region of the amygdala template as defined by the AAL brain atlas (Ashburner, 2007). We demonstrated amygdala volumetric differences between migraineurs and HC. The EMwD group had the largest amygdala volume among the three groups, and its volume was significantly larger than in the HC group. The amygdala volume in the EM group was also larger than in the HC group. There were no significant differences in amygdala volume between EMwD and EM, but the volume in the EMwD group presented a tendency to be slightly higher compared with the EM group.

Some previous studies in animal models (Gonçalves et al., 2008) or humans (Mao et al., 2013) reported that the volume of the amygdala was increased in neuropathic pain. The alterations of the amygdala volume were also reported in migraine pain. A voxel‐based morphometry data of migraine patients established that the gray matter volume (GMV) is decreased in the central pain processing structure, including the amygdala and middle and inferior frontal gyrus. The amygdala volume in chronic migraines is smaller than in EM (Valfrè et al., 2008). A previous study showed that the changes in amygdala volume were correlated with headache frequency in specific ranges (Liu et al., 2017). On the other hand, studies showed that amygdala volumes were associated with depressive symptoms, although this association was affected by many factors, such as heredity, gender, course of the disease, and age. The left amygdala GMV was larger in patients with late‐onset depression and smaller in patients with prolonged depression in one study, indicating that duration of depression is an affecting factor of GVM (Zavorotnyy et al., 2018). A significant negative relationship was observed between depressive symptomatology scores and the right amygdala volume in the 18−39 age group (Daftary et al., 2019). In the present study, the amygdala volume was increased in migraineurs with or without comorbid depression. We speculated that the change in amygdala volume is related to neuroplastic alterations induced by pain. Given the migraine headache attack be regarded as a stressor, the increase of amygdala volume indicates a brain adjustment and adaptation to pain. We did not find a significant difference in amygdala volume between migraineurs with and without depression, but the volume in the EMwD group presented a tendency of increment. Because of the complexity of the amygdala volume alterations in patients with depression, we speculate that depressive symptoms play a role in promoting the change in amygdala volume in the comorbid condition. Thus, it might be proposed that the tendency of amygdala volume increment might result from the comorbidity, and the headache attacks play a more relevant role in its injury. It should be noted that some inconsistencies in amygdala volume alterations existed between our study and previous studies. Migraine comorbid with MDD is a heterogeneous condition in which the patients will experience various extents of the symptoms of both diseases (Bergman‐Bock, 2018). Furthermore, migraine is a heterogeneous condition characterized by different frequencies and severity of attacks among patients (Leo & Singh, 2016). Therefore, it is likely that differences in the included participants among studies might lead to differences from one study to another. Furthermore, differences in fMRI protocols and analysis software might influence the results (Binder et al., 2005; Binder et al., 2008; Soares et al., 2016).

In the present study, we found a significantly decreased rsFC of the right CMA with DLPFC but decreased rsFC of the left CMA with the left pallidum in the EMwD group compared with the EM group. These different patterns of the right and left CMA FC might be related to the different functions of the bilateral amygdala. The left amygdala modulates either pleasant or unpleasant emotions, such as fear, anxiety, and sadness, while the right amygdala mainly induces negative emotions (Lanteaume et al., 2007).

The DLPFC, located in Brodmann's areas 9 and 46, regulates cognitive and executive functions and plays a vital role in MDD (Koenigs & Grafman, 2009). Previous studies reported that in MDD patients, the DLPFC exhibits abnormal metabolism (Koenigs & Grafman, 2009), blood flow reduction (Caetano et al., 2005), and GMV reduction (Chang et al., 2011). In postmortem samples from MDD patients, the density of neurons and glial cells in this area was decreased (Si et al., 2004). Therefore, the DLPFC is thought to be a key area associated with the pathophysiology of MDD. In chronic migraine, prefrontal lobe dysfunction had already been reported in the form of impaired neuropsychological tests (Mongini et al., 2005). In addition, a study involving healthy people showed that stimulation of the left DLPFC suppressed pain induced by capsaicin on the back of both hands, while stimulation to the right DLPFC did not affect the pain (Brighina et al., 2011). Concerning the condition of the comorbidity of migraine with depression, a previous magnetic resonance spectroscopy study in patients demonstrated an increased myo‐inositol/total creatine (mI/tCr) ratio in the DLPFC (Lirng et al., 2015). A transcranial magnetic stimulation (TMS) research conducted with high‐frequency repetitive TMS over the left DLPFC in migraine patients with comorbid depression demonstrated a significant reduction in headache frequency, VAS score, and impact on daily life at the end of treatment (Kumar et al., 2018). In this study, we found decreased rsFC of the right CMA with the left DLPFC in EMwD compared with that in EM but increased rsFC of the right CMA with the DLPFC in EM as compared with HC. Thus, we speculate that the bilateral DLPFC is related to the onset of migraine, and the left DLPFC is associated with the comorbidity of migraine with depression. Still, the present study was not designed to determine the pathogenic mechanisms involved in EM and EMwD, but this study could only observe the changes in both conditions. There is a possibility the additive changes of EM and MDD result in signs that are contrary to those of EM alone. Future studies will have to examine those mechanisms using both resting‐state and task fMRI, as well as the changes in electroencephalography and structural MRI.

The results revealed that EMwD exhibited significantly decreased rsFC of the amygdala compared with EM, while EM showed enhanced rsFC of the amygdala compared with HC. The decreased rsFC between CMA and DLPFC in migraineurs with depression is a key finding in this study. This disrupted connection might be a potential neuropathological basis for the coexistence of migraine and depression. A better understanding of the pathogenesis of comorbidity is helpful for the further classification and diagnosis of diseases and the optimization of clinical treatment. For example, increasing the connection between the CMA and DLPFC through deep target stimulation might provide an effective treatment for migraineurs with depression.

The study has several limitations. First, the sample size of the EMwD and EM groups was relatively small and might limit the statistical power. Second, this study was a cross‐sectional study, and it is not clear whether the amygdala volume and functional alteration could be used to predict the treatment outcomes in migraine patients comorbid with depression. Causal relationships will have to be examined in future studies. Third, this study included only patients with EM without aura. The comparison of episodic versus chronic migraine and with versus without aura will have to be performed in future studies. Fourth, our study was designed in 2018, and only the ICHD‐3 beta version was available at that time. Fifth, the restriction to one main region (the amygdala) might hinder other possible statistically significant relationships that might be disrupted in the EM and EMwD groups. Due to the lack of T2 or FLAIR acquisition, no WM hyperintensities could be assessed, at least as a covariate. Sixth, prefrontal dysfunction can be tentatively identified by some scale measures, but an MRI might be needed for confirmation, which was not performed here. Finally, the study period was partly during the COVID‐19. The COVID‐19 epidemic began in 2020, and all the fMRI in the HC group were completed in 2019. Some patients with migraines were recruited in 2020, but recruitment was stopped from February 2020 to June 2020, which was the lockdown period in China. Our province was not affected much by the COVID‐19 epidemic area, and all participants had their health codes in the later stage. People from medium‐high‐risk areas could not enter the University and were not enrolled. In addition, the temperature monitoring of the subjects was normal without respiratory symptoms.

## 5 CONCLUSIONS

It is the first study to explore the alterations of the amygdala volume and the FC in migraine with comorbid depression. This study suggests that an enlarged amygdala is an imaging feature of EM and EMwD. The inconsistency of rsFC between CMA and DLPFC between migraineurs with and without depression might indicate that decreased rsFC between CMA and DLPFC is a neuropathologic marker for the comorbidity of migraine and depression. The core regions might be a potential intervention target for the treatment of EMwD in the future.

## AUTHOR CONTRIBUTIONS

Yu Wang designed the study and revised the final manuscript. Xin Chen performed the experiments and data analysis and wrote the draft manuscript. Wei Gui, Han‐Li Li, and Zi‐Ru Deng contributed to clinical data collection and assessment. All authors read and approved the final version of the manuscript.

## FUNDING INFORMATION

This work was supported by the National Natural Science Foundation of China to Yu Wang (Grant No. 81671290) and the Foundation of Anhui Medical University to Xin Chen (No. 2019xkj155) and the Foundation of Anhui Public Health Clinical Center to Xin Chen (No. 2023YKJ02).

## CONFLICT OF INTEREST STATEMENT

On behalf of all authors, the corresponding author states that there is no conflict of interest.

### ETHICS APPROVAL AND CONSENT TO PARTICIPATE

The studies involving human participants were reviewed and approved by the Ethics Committee of The First Affiliated Hospital of Anhui Medical University. The patients/participants provided their written informed consent to participate in this study.

### PEER REVIEW

The peer review history for this article is available at https://publons.com/publon/10.1002/brb3.3427.

## Data Availability

The datasets associated with this study are available on request to the corresponding author.
